# Case Report: Filamin-C (*FLNC*) as a cause of disease in a large South African family diagnosed with restrictive cardiomyopathy

**DOI:** 10.3389/fmed.2026.1805706

**Published:** 2026-04-29

**Authors:** Polycarp Ndibangwi, Kayla Wagenaar, Nakita Laing, Jacqui Cirota, Careni Spencer, Ntobeko Ntusi, Sarah Kraus, Gasnat Shaboodien

**Affiliations:** 1 Cardiovascular Genetics Laboratory, Department of Medicine, Cape Heart Institute, University of Cape Town, Cape Town, South Africa; 2 Department of Medicine, Cardiac Clinic, University of Cape Town and Groote Schuur Hospital, Cape Town, South Africa; 3 Department of Medicine, University of Cape Town, Cape Town, South Africa; 4 South African Medical Research Council, Cape Town, South Africa

**Keywords:** disease-causing variants, familial RCM 5, filamin-C, next-generation sequencing, restrictive cardiomyopathy

## Abstract

Restrictive cardiomyopathy (RCM) is a rare cause of cardiomyopathy, the etiology of which remains poorly understood and may result from inherited or acquired predispositions to disease, or a combination thereof. Familial RCM 5 (OMIM 617047) usually has autosomal dominant inheritance with most of the identified genes encoding sarcomere or Z-disk proteins. The aim of this study was to determine the disease-causing variant in a South African family diagnosed with RCM. A South African family was screened at Groote Schuur Hospital, and the DNA of the affected individuals were investigated using next-generating sequencing methods with a custom cardiomyopathy panel of 38 genes. Possible RCM-causing variants were selected according to the ACMG/AMP guidelines. Primers were designed, and the Sanger sequencing method was used for variant validation. Segregation analysis was performed after the RCM-causing variant was identified. The family was initially diagnosed with Noonan syndrome-associated cardiomyopathy based on suggestive clinical findings; however, panel screening for Noonan’s found no causative gene. The diagnosis was revised, and an alternative cause of RCM was considered. A custom cardiomyopathy panel sequencing found a heterozygous missense NM_001458.5 (*FLNC*): c.6031G > A (p.Gly2011Arg) variant in all affected individuals. The variant is located in the FLNC protein R18 Ig-loop of the rod 2 domain and has been associated with severe RCM. This case highlights the importance of considering a broader genetic differential diagnosis in patients presenting with Noonan syndrome-like features, particularly when cardiac abnormalities are present and initial genetic testing for common Noonan syndrome genes is negative. Mutations in genes like *FLNC*, while not typically associated with classic Noonan syndrome, can cause overlapping clinical presentations. Whole exome sequencing can be a valuable tool for identifying these alternative genetic etiologies and guiding appropriate clinical management and genetic counseling for families.

## 1 Introduction

Restrictive cardiomyopathy (RCM) is a rare condition that includes a heterogeneous group of myocardial diseases that result in increased myocardial stiffness, leading to impaired ventricular filling and diastolic dysfunction. RCM can be diagnostically challenging due to its varied pathogenesis, clinical presentation, and diagnostic evaluation (1). Also, RCM is the least common type of cardiomyopathy.

In general, genetic RCM is characterized by near normal-sized left ventricle with enhanced ventricular stiffness, resulting in impaired ventricular filling, increased end-diastolic pressure, and atrial enlargement. Early in the disease, systolic function may be normal, or mildly impaired, with significant systolic impairment usually only occurring in end-stage disease. Mild left ventricular hypertrophy may be present, which can make it difficult to differentiate from hypertrophic cardiomyopathy (HCM), especially in the early stages of the disease. In addition, patients with HCM may have “restrictive physiology” with impaired ventricular filling and atrial enlargement, although usually to a lesser degree (2).

Restrictive cardiomyopathy presents unique clinical challenges due to the progressive nature of the disease and the limited treatment options available. In Africa, endomyocardial fibrosis, and factors such as genetic predisposition, infectious diseases, and environmental influences might play significant roles in the disease’s manifestation. The prevalence of RCM in Africa is unknown and there is a critical need for comprehensive research to understand its prevalence, etiology, and optimal management strategies in this region (3). The aim of this study was to determine the genetic cause of disease in a family with a distinctive cardiac phenotype.

## 2 Case description

**FIGURE 1:**
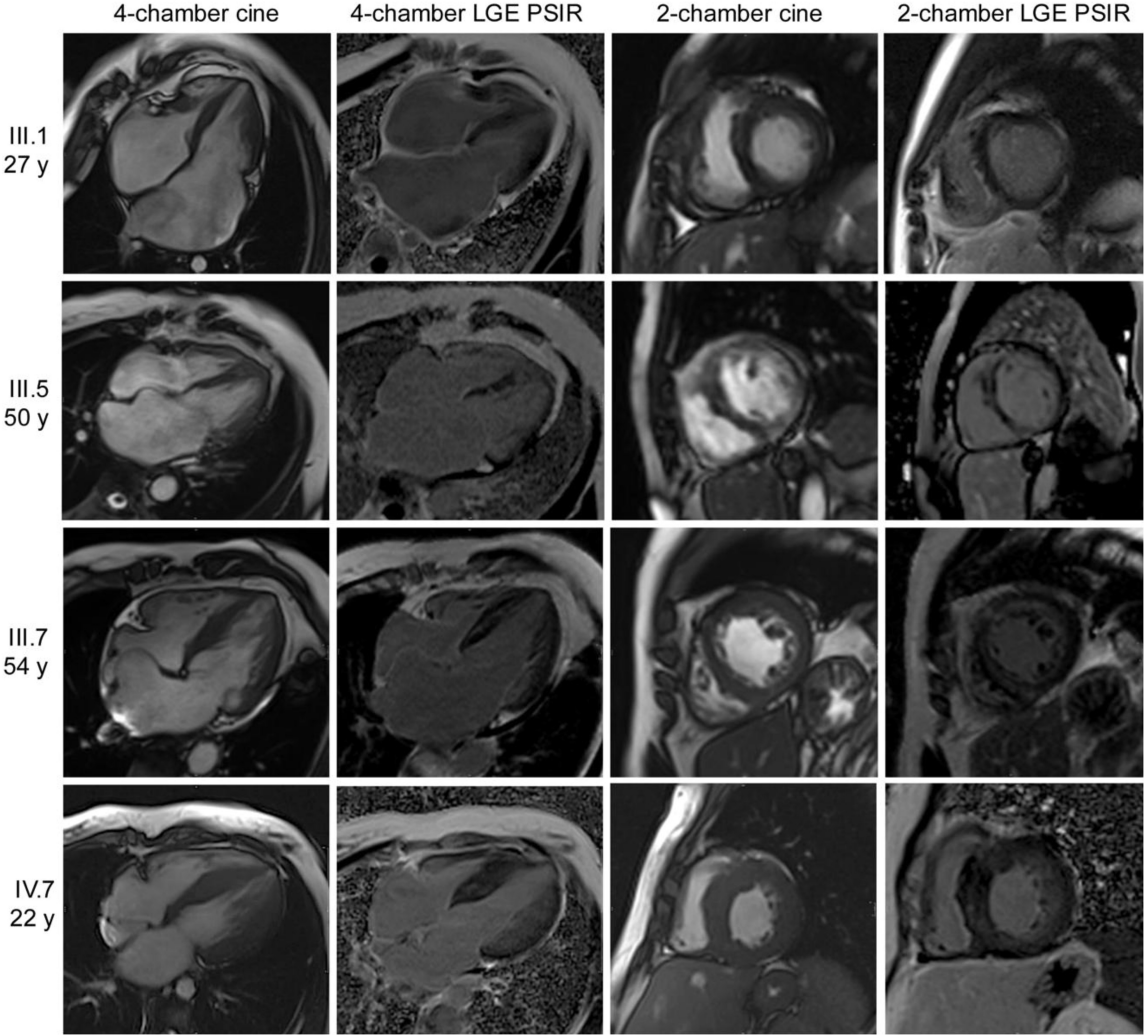
Cardiac magnetic resonance (CMR) images of proband (III.1) and clinically affected family members (III.5, III.7 and IV.7).

The proband (III.1) was diagnosed with heart failure (HF) and showed features of RCM at age 27 years (Figure 1). Unfortunately, due to the presence of significant irreversible pulmonary hypertension, he was not amenable to cardiac transplantation. He developed end-stage HF within 2 years of presentation and died while hospitalized with community-acquired pneumonia at the age of 32. In light of a family history of cardiomyopathy in the proband’s father (II.2 - deceased), all first-degree relatives were invited for family screening. The proband’s mother (II.1), brother (III.2) and half-brother (III.3) were noted to be clinically unaffected. The proband’s half-sisters, III.5 (43 years) and III.7 (42 years) both had features of early cardiomyopathy with mild atrial dilatation and a history of atrial tachyarrhythmias. Individual IV.7 was 22 years of age at the time of initial screening and was the first person diagnosed with possible Noonan’s syndrome in this family. She reported cardiac symptoms including palpitations, chest pain and effort intolerance. She was noted to have prominent “syndromic features” (short stature, broad neck, easily bruising, café au lait spots, a prominent sternum and severe scoliosis) and features suggestive of an HCM phenocopy with concentric left ventricular hypertrophy, diastolic dysfunction and moderately elevated left ventricular end-diastolic pressure on echocardiography. On further review of other family members, in addition to the cardiac findings, multiple individuals were thought to have “Noonan-like” characteristics. The family was referred to medical genetics for review and genetic testing. However, genetic testing for Noonan’s syndrome for IV.7 found no causative genetic variant and the primary diagnosis was revised. Alternative genetic causes of RCM were considered, and the baseline characteristics, genetics and outcome findings are shown in Table 1.

**TABLE 1:** Baseline characteristics at time of diagnosis/screening, genetics and outcomes of proband and relatives.

Patient	Sex	Age (yrs)	*FLNC* variant	Cardiac phenotype	Other clinical features	Arrhythmia	LAD (mm)/LAA (cm^2^)	MWT (mm)	LVEF %	Restrictive ventricular filling	Age at follow-up (yrs)	Outcome at follow-up
II.1	F	58	Negative	Unaffected	–	No	30/–	10.3	60	No	70	Alive/unaffected
II.2	M	21	No DNA available	CMO unspecified	Unknown	Unknown	–	–	–	–	60	Died
Proband III.1	M	27	*FLNC* c.6031G > A	RCM	Scoliosis	AF	53/33	10.2	48	Yes	32	Died
III.2	M	32	Negative	Unaffected	–	No	29/14	8.5	74	No	43	Alive/unaffected
III.3	M	46	Negative	Unaffected	–	No	34/–	8.5	66	No	58	Alive/unaffected
III.5	F	43	*FLNC* c.6031G > A	RCM	Short, broad neck, scoliosis	AF	44/28	9.2	38	Yes	55	Alive/OHT at age 50
III.7	F	42	*FLNC* c.6031G > A	Early RCM	Short, epicanthic fold, easy bruising	Atrial flutter	37/28	11	47	No	54	Alive/affected
IV.1	M	32	Negative	Unaffected	Café au lait	No	29/–	9.7	68	No	32	Alive/unaffected
IV.3	M	29	*FLNC* c.6031G > A	Early RCM	Broad neck	No	41/–	9.9	62	Borderline	30	Alive/asymptomatic
IV.5	M	18	Negative	Unaffected	–	–	–	–	–	–	18	Alive/unaffected
IV.6	M	16	*FLNC* c.6031G > A	Unaffected carrier	Broad neck	No	Normal	8.6	83	No	16	Alive/asymptomatic carrier
IV.7	F	22	*FLNC* c.6031G > A	RCM (HCM overlap)	Short stature, broad neck, scoliosis, easy bruising, café au lait	Atrial flutter (onset at 33 years)	41/28	17	63	Yes	34	Advanced disease, awaiting OHT
V.2	F	7 mth	*FLNC* c.6031G > A	RCM with multiple VSDs	Lymphoedema, dysmorphism	No	Dilated	4	72	Unknown	4	Alive/affected

AF, Atrial flutter; mth, months; M, Male; F, Female; *FLNC*, Filamin-C; CMO, cardiomyopathy; RCM, restrictive cardiomyopathy; LAD/LAA, left atrial dimension/left atrial area; MWT, maximal wall thickness; LVEF, left ventricular ejection fraction; OHT, orthotopic heart transplant; VSDs, ventricular septal defects; “–,” not recorded. Age-dependent penetrance anticipated; ongoing guideline-directed clinical surveillance recommended.

**FIGURE 2:**
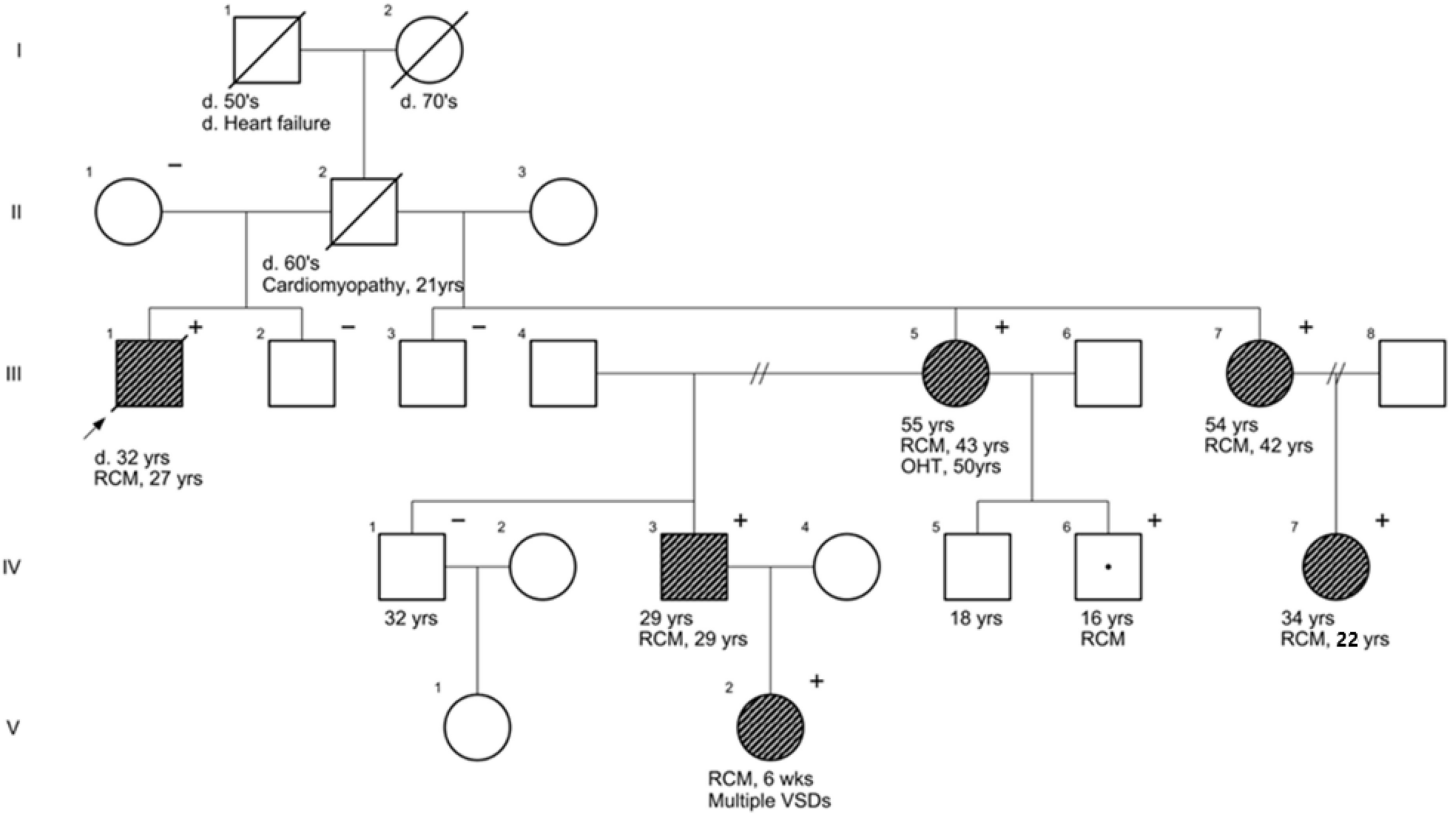
Pedigree of family members showing segregation of *FLNC*: c.6031G > A (p.Gly2011Arg), NM_001458.5 with clinical disease. The arrow indicates the proband and the black shading indicates affected individuals. Open symbol with “+” indicates genotype-positive, currently asymptomatic (age-dependent penetrance). OHT, orthotopic heart transplant; RCM, restrictive cardiomyopathy; VSDs, ventricular septal defects.

We performed panel sequencing of 38 cardiomyopathy-associated genes and a heterozygous missense variant in the filamin-C (*FLNC*) gene was identified. This *FLNC*: c.6031G > A (p.Gly2011Arg) variant in exon 37 (NM_001458.5; ENST00000325888.8) occurred in a known hotspot in the R18 Ig-loop of the ROD2 domain. The variant was detected in all four clinically affected relatives screened (III.5, III.7, IV.3, and IV.7) (Figure 2). The remaining clinically unaffected family members (II.1, III.2, III.3, and IV.1) tested negative for the c.6031G > A variant and were discharged from follow-up. Family members IV.5, IV.6 and V.2 were offered predictive genetic testing, and the following outcomes were observed: individual IV.5 tested negative for the familial variant, thus eliminating the need for further clinical screening. Conversely, individual IV.6 tested positive and, despite being asymptomatic, will be offered guideline-directed clinical screening to monitor for any potential disease manifestation. Individual V.2 had an existing diagnosis of multiple small ventricular septal defects prior to genetic testing, having presented with heart failure in infancy.

## 3 Diagnostic assessment (methods)

A South African family with familial restrictive cardiomyopathy was recruited into the African Cardiomyopathy and Myocarditis Registry Program (IMHOTEP) at Groote Schuur Hospital in Cape Town, South Africa (4). A family pedigree was constructed at the time of the proband’s initial consultation, and “at-risk” family members were invited for screening. Clinical phenotyping was conducted on all available family members. Baseline cardiac investigations including electrocardiograms and echocardiography were performed on all individuals. Extended investigations such as cardiovascular magnetic resonance (CMR), 24-h Holter, angiography and endomyocardial biopsies were performed on affected individuals according to clinical indications. All individuals provided written informed consent for enrolment into IMHOTEP (HREC 766/2014), and blood samples were collected for next-generation sequencing (NGS).

Blood samples were collected from nine family members; of which four were clinically affected. Genomic DNA was extracted and prepared for NGS. We amplified the gDNA and performed a targeted NGS panel of 38 cardiomyopathy-associated genes using the Twist Library Preparation Kit (Twist Bioscience HQ). The targeted exons were sequenced at the Oxford Genomics Center (Wellcome Center for Human Genetics, University of Oxford, UK) using a NovaSeq 6000 sequencer (Illumina Inc.). Variant calling was performed on the NGS data using the Illumina platform and the output was stored as VCF files.

The VCF files annotation were achieved using computational facilities provided by the University of Cape Town’s ICTS High-Performance Computing (HPC) team. The variant annotation pipeline (Supplementary Figure 1) was run on the HPC cluster using Ensembl Variant Effect Predictor (VEP) release 107 (5). Variants such as insertions, deletions, missense variants, nonsense variants and splice donor variants were included; synonymous variants were excluded. Variants were also filtered against the Genome Aggregation Database (gnomAD v2.1) with minor allele frequencies (MAF) of ≤0.01 considered as rare. The variants were further investigated and categorized according to the American College of Medical Genetics and Genomic (ACMG) (6) guidelines.

The 13 points ACMG criteria for this c.6031G > A were PP5 Very Strong, PP3 Strong (e.g., Align GVGD: Class C65, CADD: 27.9, Mutation taster: Deleterious, PolyPhen2: Probably damaging, SIFT: Deleterious), PP1 Moderate, PM1 Moderate and PM2 Supporting. The pathogenicity of the filtered variants was predicted using Alamut™ Visual Plus (SOPHiA GENETICS; version 1.10) with the built-in software modules ClinVar, Mastermind, SIFT, PolyPhen HDIV, PolyPhen HVAR, Mutation Taster, FATHMM, CADD13, DANN, M-CAP, and REVEL, as well as using the ACMG manual. To confirm the presence of the final filtered variant, primers were designed, synthesized and variant validated using Sanger sequencing (Supplementary Figure 2).

## 4 Discussion

We describe a South African family where an initial diagnosis of Noonan syndrome-associated cardiomyopathy was considered based on suggestive clinical findings; however, panel screening for Noonan’s found no causative gene. The diagnosis was revised, and an alternative cause of RCM was considered.

We performed NGS using a custom cardiomyopathy panel of 38 genes and found a heterozygous missense c.6031G > A variant in the ROD2 domain of *FLNC* gene in all affected family members. Per the ACMG guidelines, this c.6031G > A variant was classified as a pathogenic variant. The c.6031G > A variant is reported in European and North American cohorts and was used to generate iPSC lines however, this is the first case reported in a South African family of Mixed Ancestry.

Filamin-C is a known causative gene in RCM patients with this particular affected amino acid (7, 8) previously reported as disease-causing in patients with RCM (8–10). *FLNC*’s main function is the binding of actin rods in the adherens junction, which are the structures that, together with desmosomes, contribute to the maintenance of the cellular integrity and force transduction of tissues exposed to mechanical stress (11).

Filamin-C mutations cause various cardiomyopathies through distinct mechanisms depending on their location within the gene. The protein interacts with key cardiomyopathy-associated proteins at the Z-disk, particularly in the actin-binding ROD2 domain. Pathogenic variants, often found in ROD1 and ROD2, disrupt these interactions, leading to protein aggregation, sarcomere disarray, and ultimately, cardiac dysfunction. The ROD2 domain, especially the conserved 18–21 cluster, is crucial for protein binding, mechanosensing, dimerization, and phosphorylation, highlighting its importance in maintaining proper cardiac muscle structure and function. Most of the pathogenic variants in *FLNC* that lead to cardiomyopathy are localized to the actin-binding domains ROD1 and ROD2, which are considered hotspots (11, 12). Variants associated with RCM and HCM are predominantly missense variants, which cluster in the ROD2, which is essential for filamin C dimerization, Z- disk interaction and cell-signaling (13). These variants interfere with the secondary protein structure, leading to sarcomere disarray and aggregate formation. These histopathological changes are associated with HCM and RCM phenotypes. Both HCM and RCM patients have an enrichment of missense variants causing changes in the secondary protein structure resulting in an abnormal protein (13). As in the current report where a mixed RCM/HCM cardiac phenotype was found, previous research by Bermudez-Jimenez et al. (12) described a distinctive cardiac phenotype characterized by severe HCM/RCM and an unusual saw-tooth left ventricular hypertrabeculation, which was associated with the ROD2 domain of *FLNC*. This overlapping HCM and RCM phenotype was previously reported in the context of mutations in cardiac sarcomere protein genes (7). They observed that most individuals had heart failure (HF) symptoms, marked diastolic dysfunction, and a high incidence of major clinical events. This was in line with a more severe form of cardiomyopathy than classic HCM, and similar to HCM, due to mutations in thin filament protein genes such as troponin T (11). They hypothesize that this finding might reveal that restrictive physiology and advanced HF in *FLNC*-mRod2 variants may be common at early stages of the disease, similarly to previous descriptions of patients with advanced HF at young ages (7, 12, 14).

In this family, there were also several non-cardiac clinical characteristics noted in affected individuals, including scoliosis. This clinical finding may be unrelated or could represent subtle axial myopathy with weakening of the erector spinae muscles. Creatinine kinase was not routinely measured in all individuals, however, in those where it was done, the levels were normal or only mildly elevated. Clinical examination did not indicate overt limb girdle weakness, however, myofibrillar myopathy could not be definitively excluded as electromyography was not available. Although paraspinal muscle weakness and limb-girdle involvement has been described in other FLNC-associated myopathies (15), it has not previously been reported in association with this specific variant (c.6031G > A) (8). While individual IV.7 did have several features consistent with Noonan’s syndrome (including scoliosis, short stature, hypertrophic cardiomyopathy), she did not have pulmonic stenosis or the typical facial features described in Noonan’s syndrome. While rare, concomitant congenital heart defects have been described in childhood onset of *FLNC*-related RCM, with ventricular septal defects (VSDs) reported in 33% of cases in one series (16).

Case IV.6, presented as an asymptomatic carrier of the pathogenic *FLNC* variant at age 16 years. This individual illustrates age-dependent penetrance, a phenomenon well-recognized in *FLNC*-associated cardiomyopathies. In these cases, the onset of disease symptoms can be delayed until the third to fifth decade of life, even within families sharing the identical variant. Ongoing monitoring is planned for case IV.6 (and other relatives who might have a positive genotype later on), which include periodic echocardiography and CMR imaging.

In conclusion, this family highlights the complexity of *FLNC*-associated conditions where cardiac phenotypes and age of onset may vary considerably, even within a family. Concomitant myofibrillar myopathy may be subtle and overlooked in patients presenting with cardiac disease, and children may present with co-existing congenital heart defects. This case underscores the need for comprehensive evaluations by multidisciplinary teams with expertise in adult and pediatric cardiology, medical genetics, and neurology, and emphasizes the value of broader genetic testing approaches in complex cases.

## Data Availability

The datasets presented in this study can be found in online repositories. The names of the repository/repositories and accession number(s) can be found below: ClinVar (Accession number SCV007579166).
