# Supplementary figures and images for: Case Report: Filamin-C (FLNC) as a cause of disease in a large South African family diagnosed with restrictive cardiomyopathy
Source: Front Med (Lausanne). 2026 Apr 29;13:1805706. doi: 10.3389/fmed.2026.1805706 (PMC13169734; doi:10.3389/fmed.2026.1805706)

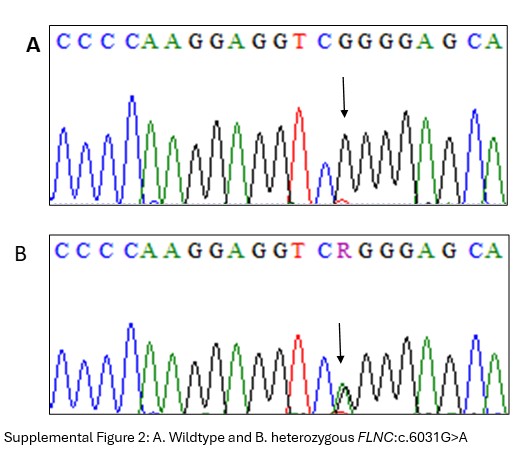

Supplement: Supplementary file 1 [file Image_1.jpeg]

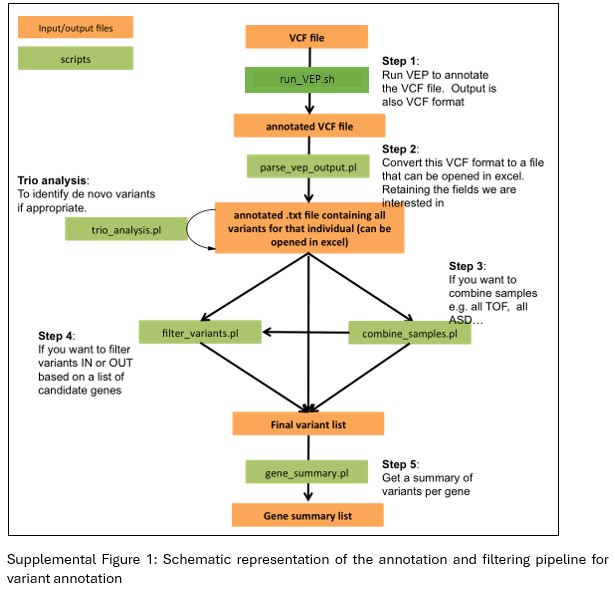

Supplement: Supplementary file 2 [file Image_2.jpeg]
